# Creating a sustainable brain health navigator model to improve the diagnostic journey for Alzheimer's disease: the experience of one health care system in Ohio

**DOI:** 10.1002/alz70860_101321

**Published:** 2025-12-23

**Authors:** Rhonna Shatz, Katherine Schmidt, Anne Paul, Elizabeth Hente, Candace Burch

**Affiliations:** ^1^ University of Cincinnati Health, Cincinnati, OH, USA

## Abstract

**Background:**

Primary care providers (PCPs) often rely on Medicare Annual Wellness Visits or patient/care partner concerns to identify Alzheimer's disease (AD), capturing only about 50% of UC Health's expected cases of mild cognitive impairment (MCI) or dementia. Multifactorial and modifiable contributors to cognitive decline, especially in racially and socioeconomically diverse populations, require more systematic and culturally appropriate evaluations. A survey of PCPs at UC Health revealed knowledge gaps in clinical syndromes, biomarkers, comorbidities, and new therapies, as well as a lack of scheduling support, diagnostic tools, and post‐diagnosis resources. Primary care residents lack opportunities to incorporate updated diagnostic and treatment protocols to assess cognitive impairment. The Brain Health Navigator (BHN) model pilot tests boundary spanning roles to streamline and simplify the diagnostic and treatment journey for both patients and providers.

**Method:**

The BHN pilot engages 10 PCPs and 41 residents in the Primary Care Network with >80 PCPs prepared to subsequently engage. The BHN serves as a central node, linking pre‐diagnosis referral with post‐visit continuing care. Key innovations include MyChart for pre‐visit screening, digital cognitive assessments by medical assistants, Epic smart sets for MRI, biomarkers, FDG‐PET, and lab orders, provider smart forms and templates, and longitudinal tracking from pre‐diagnosis to continuing care. An AI‐driven “staff double” is being tested to enhance completion of electronic questionnaires and improve patient understanding before clinic visits.

**Result:**

In collaboration with the Alzheimer's Association, UC Health hired a social worker to gather functional and behavioral data prior to the visit and to offer families ongoing support. By offloading administrative and care coordination tasks to the BHN and the social worker, PCPs can focus on clinical interpretation and examination, improving patient‐centered care and overall diagnostic accuracy.

**Conclusion:**

The BHN model demonstrates how boundary‐spanning roles and integrated workflows can support timely, accurate diagnoses of AD within primary and specialty care. This approach, backed by DAC‐SP resources, holds promise for scaling to other health systems aiming to enhance the cognitive diagnostic process and patient experience.

